# Stability across time of the neutrophil-lymphocyte and lymphocyte-neutrophil ratios and associations with outcomes in cardiac surgery patients

**DOI:** 10.1186/s13019-019-0988-6

**Published:** 2019-09-11

**Authors:** Jeremy Green, Syed Usman Bin Mahmood, Makoto Mori, Sameh Yousef, Abeel A. Mangi, Arnar Geirsson

**Affiliations:** 1 Frank H. Netter School of Medicine at Quinnipiac University, North Haven, USA; 2 Section of Cardiac Surgery, Yale University School of Medicine, New Haven, USA

**Keywords:** Cardiac surgery, Risk prediction, Inflammation, Neutrophil-lymphocyte ration

## Abstract

**Background:**

Elevated white blood cell counts and leukocyte ratios are recognized markers of systemic inflammation associated with poor surgical outcomes. We analyzed the temporal stability and prognostic value of the preoperative Neutrophil-to-Lymphocyte ratio (NLR) and Lymphocyte-to-Neutrophil ratio (LNR) in patients undergoing coronary artery bypass grafts and/or valve surgery.

**Methods:**

We retrospectively reviewed 558 patients who underwent elective cardiac surgery between July 2014 and December 2016, excluding patients with immunosuppressed status. The stability of NLR and LNR was evaluated using interclass correlation coefficients. The patients were dichotomized into two groups, high NLR and low NLR, based on the median value of preoperative NLR in our cohort. A logistic regression model compared preoperative NLR and LNR values to clinical outcomes, including a composite of perioperative events and mid-term mortality.

**Results:**

We identified that NLR reliability over time was good (ICC = .592; R-squared = .351), and LNR reliability over time was excellent (ICC = .761; R-squared = .601). Furthermore, an increased duration between collection dates was not significantly correlated with increased variability in NLR (Pearson Correlation Coefficient: −.111, *p* = .117). On multivariate model, neither high NLR (OR = .879, *p* = .168) nor low LNR (OR = 3.30, *p* = .214) was significantly associated with a composite of perioperative events, but increased LNR was associated with lower mid-term mortality (HR .001, *p* = .026).

**Conclusions:**

Both NLR and LNR were stable over time, up to 100 days, but LNR values were more consistent compared to NLR. High LNR was significantly associated with decreased risk of mid-term mortality, and LNR showed a stronger relationship with mid-term mortality than its NLR counterpart. Both of these findings indicate that LNR may be a more useful and valuable clinical marker.

## Introduction

Increased white blood cell counts are used as a generalized marker of inflammation and as a predictor of morbidity and mortality after surgery [1, 2]. However, the value of absolute white cell counts is limited due to variation between different individuals, variation within a given patient based on their health status or environment at the time of collection, and inconsistent cell classification or sample preparation [3, 4]. As a result, the Neutrophil-to-Lymphocyte ratio (NLR) has emerged as an alternative predictor of mortality and morbidity in various areas of surgery [1, 5]. The NLR integrates the detrimental effects of both neutrophilia, reflecting inflammation, and lymphopenia, a marker of poorer general health and physiologic stress [6]. Its inverse, the Lymphocyte-to-Neutrophil ratio (LNR), serves the same purpose. In recent studies, NLR has been shown to be more strongly associated with mortality than the absolute white cell count in patients undergoing coronary artery bypass grafts [4].

Although they are potentially useful markers, the temporal stability of NLR or LNR values has not been previously studied. Therefore, our understanding of their reliability is limited, since the measurements could vary significantly depending on when the measurements take place relative to the date of operation. Understanding the temporal stability of leukocyte ratios is critical for assessing their clinical utility in improving preoperative risk assessment. In addition, defining a window of time where the values are stable and predictive of postoperative events would greatly enhance their clinical utility.

In this study, we sought to investigate the stability of NLR and LNR over time and evaluate them as potential predictors of outcomes following cardiac surgery in patients undergoing Coronary Artery Bypass Grafts (CABG) and valve operations at our center.

## Patients and methods

### Patients

A retrospective cohort study was performed including patients undergoing isolated CABG, isolated valve surgery, or a combination of the two at Yale-New Haven Hospital between July 2014 and December 2016. Initial screening yielded 2206 patients. We excluded patients with non-elective operative status, as well as those who did not have a complete blood count (CBC) with differential values recorded prior to their operation or had lab values that were collected over 60 days before the date of surgery. Patients were also excluded in cases of active endocarditis or documented immunosuppressed status, which includes immunosuppressive condition (HIV, hypogammaglobulinemia, splenectomy, etc.) or medication (systemic steroid therapy, anti-rejection medications, chemotherapy, etc.) use as outlined in the STS Adult Cardiac Surgery Database Training Manual (Version 2.81) [7]. The final cohort for the analysis consisted of 558 patients. In order to evaluate the stability of granulocyte measures across time on an individual patient level, an additional set of absolute neutrophil and lymphocyte values were extracted between 8 and 99 days before the first measurement for 201 of the patients who had multiple CBC measurements available. As the purpose of this portion of the study was to evaluate the stability of measurements over time, this time frame was defined in order to avoid inflation of concordance due to consecutive measures obtained in close proximity (i.e. daily lab draws). In cases where multiple CBC values were available within the defined time range, the CBC closest to the date of surgery was always used in order to calculate pre-operative NLR and LNR. The cohort was dichotomized by the median value of NLR in order to compare clinical characteristics and associations with post-operative outcomes.

### Data and outcomes

Baseline demographic and comorbidity data and operative and post-operative data were collected. The Society of Thoracic Surgeons data definitions (version 2.81) were used (https://www.sts.org/registries-research-center/sts-national-database/adult-cardiac-surgery-database/data-collection). Estimated glomerular filtration rate (eGFR) was calculated using MDMR equation [8] based on the last available creatinine values prior to the surgery.

The evaluated outcomes were in-hospital mortality and post-hospital survival since the time of surgery. Mid-term survival was evaluated up to 2 years, although 2-year follow up data was incomplete due to a proportion of patients undergoing surgery within 2 years of the last follow up date. Post-discharge survival was ascertained by linking the institutional patient-level data to Connecticut State Department of Public Health death records, which captures all mortality that occurred in the population of Connecticut state residents. The final date of follow-up was December 31st 2016. Postoperative complications were collected up until the time of hospital discharge.

### Statistical analysis

Previous studies have focused on the use of NLR as a matter of convention, but the prioritization of NLR over LNR has no clear rational given our analysis of the literature. A preliminary analysis of our data indicated that LNR data was less influenced by outliers and potentially more consistent over time, so we decided to add LNR to our statistical analysis in order to evaluate its value as a potential alternate predictor to NLR.

The temporal stability of NLR and LNR was evaluated by calculating the interclass correlation coefficient (ICC). The cutoff value for dichotomization as high or low NLR was based on the median value in our cohort. Logistic regression was used to identify predictors of outcomes. Variables evaluated as predictors of outcomes included both NLR and LNR. There was no attempt to manipulate the number of days between CBC measurements nor the NLR cutoff value. The acceptable number of days between measurements (between 8 and 99 days) was selected arbitrarily with consideration of clinical utility of the time period and the preservation of sample size for our analysis. The range was also purposefully selected to extend past the range used to analyze the relationship between NLR, LNR and outcomes (up to 60 days). The NLR cutoff value used to dichotomize the group was determined based on the median within our sample in order to create cohorts of relatively equal size. Other cutoff values were not tested and no attempt was made to maximize statistical significance by manipulating this factor.

Differences in the patient characteristics between those with NLR values above and below the median cutoff were compared with two-tailed t-test for continuous variables, chi-square test for categorical variables, or Fisher’s exact test for categorical variables with cell count ≤5. Continuous variables were summarized by mean with standard deviation, unless otherwise specified. Categorical variables are summarized by percentages. Mid-term survival relationship between time-to-death and NLR or LNR was modeled using semiparametric Cox proportional hazard model. Assuming rare incidences of the outcome, odds ratio derived from models are assumed to approximate relative risk and referred to as risk of death [9]. A *p*-value of < 0.05 was used to define statistically significant differences and correlations. All analysis was conducted with SAS 9.4 (SAS Institute Inc., Cary, NC). We framed our cohort analysis in terms of NLR, consistent with past research. However, the same high NLR and low NLR cohorts correspond to low LNR and high LNR respectively, based on the inverse relationship between NLR and LNR.

### Definitions

Patients were dichotomized into two groups based on their NLR values. The patients categorized as having high NLR were defined as having NLR > 2.44 and the patients categorized as having low NLR were defined as having NLR ≤2.44. These same cohorts also define patients with a low LNR as having LNR < .41 and high LNR as having LNR ≥ .41. The following definitions were used for the postoperative complications: postoperative prolonged intubation as mechanical ventilation > 24 h, postoperative renal failure as either new dialysis requirement in the postoperative period, increase in serum creatinine level 3 times greater than baseline, or postoperative serum creatinine level ≥ 4 mg/dL, and stroke as neurological deficit of abrupt onset caused by a disturbance in blood supply to the brain that did not resolve within 24 h. Mid-term survival was evaluated up to 2 years following the date of surgery.

## Results

### Stability over time

**Fig. 1:**
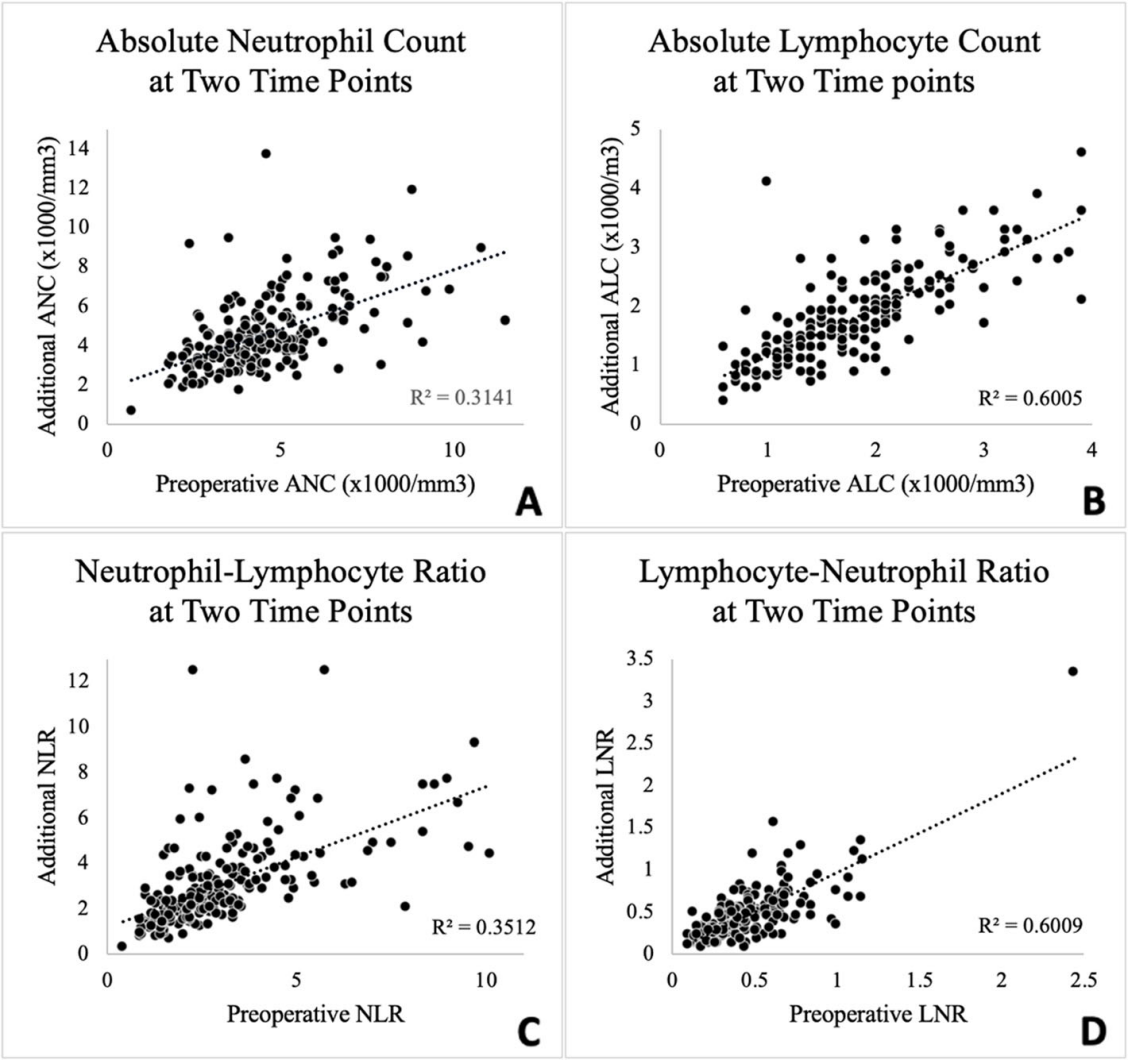
Charting ANC, ALC, NLR, and LNR at two different time points .ANC = Absolute Neutrophil Count, ALC = Absolute Lymphocyte Count. NLR = Neutrophil to Lymphocyte Ratio, LNR = Lymphocyte to Neutrophil Ratio. The preoperative measurement refers to the CBC collected nearest to the date of surgery. The additional measurement refers to a prior CBC collected between 8 and 99 days before the preoperative measurement. **a** Charting Absolute Neutrophil Count at Two Time Points. **b** Charting Absolute Lymphocyte Count at Two Time Points. **c** Charting Neutrophil to Lymphocyte Ratio at Two Time Points. **d** Charting Lymphocyte to Neutrophil Ratio at Two Time Points

Based on the 95% confidence interval of the ICC estimate, the reliability of NLR values over time was good (ICC = .592, CI = .502–.682, *p* < .001; R-squared = .351). Furthermore, an analysis of the percent change in NLR between readings and the time between those measurements found that an increased duration between readings was not significantly correlated with increased variability (Pearson Correlation Coefficient: −.111, 95%CI: −.245–.028, *p* = .117). Based on the 95% confidence interval of the ICC estimate, the reliability of LNR values over time was excellent (ICC = .761, CI = .702–.819, *p* < .001; R-squared = .601). Graphs of NLR and LNR, as well as absolute neutrophil and lymphocyte counts, measured at two different time points can be seen in Fig. 1.

### Baseline patient characteristics

The cutoff value for high NLR was set above the median value of 2.44, while patients with LNR ≤ 2.44 were considered to have low NLR. Baseline patient characteristics and operative data are shown in Tables 1 and 2. By univariate analysis, patients in the high NLR group had a higher incidence of comorbidity with lower renal function (eGFR, *p* = .0003; low NLR: mean = 88.7 ml/min/1.73m^2^, SD = 28.9; high NLR: mean = 79.7 ml/min/1.73m^2^, SD = 29.1) and congestive heart failure (*p* = .0011; low NLR: 21.4%; high LNR 33.8%), as well as a higher percentage of patients with previous sternotomy (*p* = .0146; low NLR: 5.7%; high NLR: 11.5%).

**Table 1:** Baseline characteristics in each group

Variable	Low NLR (*N* = 280)	High NLR(*N* = 278)	*p*-Value
Age	64.7 ± 11.5	68.4 ± 11.4	0.0001
Gender- Female	101 (36.1)	84 (30.2)	0.1508
Race			0.0033
Caucasian	230 (82.1)	255 (91.7)	
African American	20 (7.1)	5 (1.8)	
Asian	7 (2.5)	4 (1.4)	
Others	23 (8.2)	14 (5)	
BMI	29.0 ± 5.3	29.6 ± 6.7	0.2764
Diabetes			0.1713
Non-insulin dependent	62 (22.1)	68 (24.5)	
Insulin-dependent	20 (7.1)	31 (11.2)	
Dyslipidemia	219 (78.2)	225 (80.9)	0.4629
eGFR (ml/min/1.73 m*2)	88.7 ± 28.9	79.7 ± 29.1	0.0003
Preoperative dialysis	1 (.4)	6 (2.2)	0.0679
Hypertension	232 (82.9)	233 (83.8)	0.8205
History of endocarditis	5 (1.8)	5 (1.8)	0.9909
Chronic lung disease	5 (1.8)	10 (3.6)	0.2027
Peripheral vascular disease	22 (7.9)	29 (10.4)	0.2913
History of stroke	15 (5.4)	14 (5)	0.8643
Prior myocardial infarction	69 (24.6)	84 (30.2)	0.1401
Redo sternotomy	16 (5.7)	32 (11.5)	0.0146
CHF	60 (21.4)	94 (33.8)	0.0011

Values are n (%) or mean ± standard deviation

*NLR* Neutrophil to Lymphocyte Ratio, *BMI* Body Mass Index eGFR: estimated glomerular filtration rate, *CHF* congestive heart failure

**Table 2:** Distribution of operative procedures in each group

Variable	Low NLR (*N* = 280)	High NLR (*N* = 278)	*p*-value
Operative Data			
Isolated CABG	102 (36.4)	78 (28.1)	0.0344
AVR	98 (35.0)	119 (42.8)	0.0586
MVR	87 (31.1)	93 (33.5)	0.5473
TVR	9 (3.2)	11 (4.0)	0.6371
CPB time (min)	101.3 ± 36.9	110.6 ± 39.3	0.0058
Leukocyte-related values			
Lymphocyte	2.21 ± 0.63	1.50 ± 0.51	<.0001
Neutrophil	3.79 ± 1.14	5.38 ± 1.82	<.0001
NLR	1.76 ± 0.43	3.83 ± 1.60	<.0001
LNR	0.62 ± 0.21	0.29 ± 0.08	<.0001

Values are n (%) or mean ± standard deviation

*NLR* Neutrophil to Lymphocyte Ratio, *LNR* Lymphocyte to Neutrophil Ratio, *CABG* Coronary Artery Bypass Graft, *AVR* Aortic Valve Replacement, *MVR* Mitral Valve Replacement, *TVR* Tricuspid Valve Replacement, *CPB* Cardiopulmonary Bypass

### Complications

The high NLR group was found to be associated with increased cardiopulmonary bypass time (*p* = .0058; low NLR: mean = 101.3 in, SD =36.9 min; high NLR: mean = 110.6 min, SD = 39.3 min) and ICU length of stay (*p* = .0127; low NLR: mean 2.98 days, SD 1.70 days; high NLR: mean = 3.38 days, SD = 2.05 days). A one-unit increase in NLR was associated with a + .13 day longer stay in the ICU (95%CI: .027–.235, *p* = .014). A one-unit increase in LNR was associated with a − .83 day (shorter) stay in the ICU (95%CI: − 1.551 - -.106, *p* = .025). Neither high NLR (*p* = .1678, OR = 0.88, 95%CI = 0.73–1.06) nor low LNR (*p* = .2143, OR = 3.30, 95%CI = 0.50–21.79), which would both indicate poorer overall health, was associated with increased overall post-operative complications during hospital stay. A summary of postoperative complications can be found in Table 3.

**Table 3:** Postoperative complication compared in high and low LNR groups

Variable	Low NLR (N = 280)	High NLR (N = 278)	*p*-Value
Reoperation for bleeding	2 (0.7)	6 (2.2)	0.1754
Stroke	2 (0.7)	1 (0.4)	1.0000
Prolonged ventilation	7 (2.5)	14 (5.0)	0.1155
Pneumonia	8 (2.9)	10 (3.6)	0.6208
ICU Length of stay	2.98 ± 1.70	3.38 ± 2.05	0.0127
In-hospital mortality	0 (0)	0 (0)	–
Any complications or death	14 (5.0)	24 (8.6)	0.0885

Values are n (%) or mean ± standard deviation

*NLR* Neutrophil to Lymphocyte Ratio, *ICU* Intensive Care Unit

### Mid-term survival

**Fig. 2:**
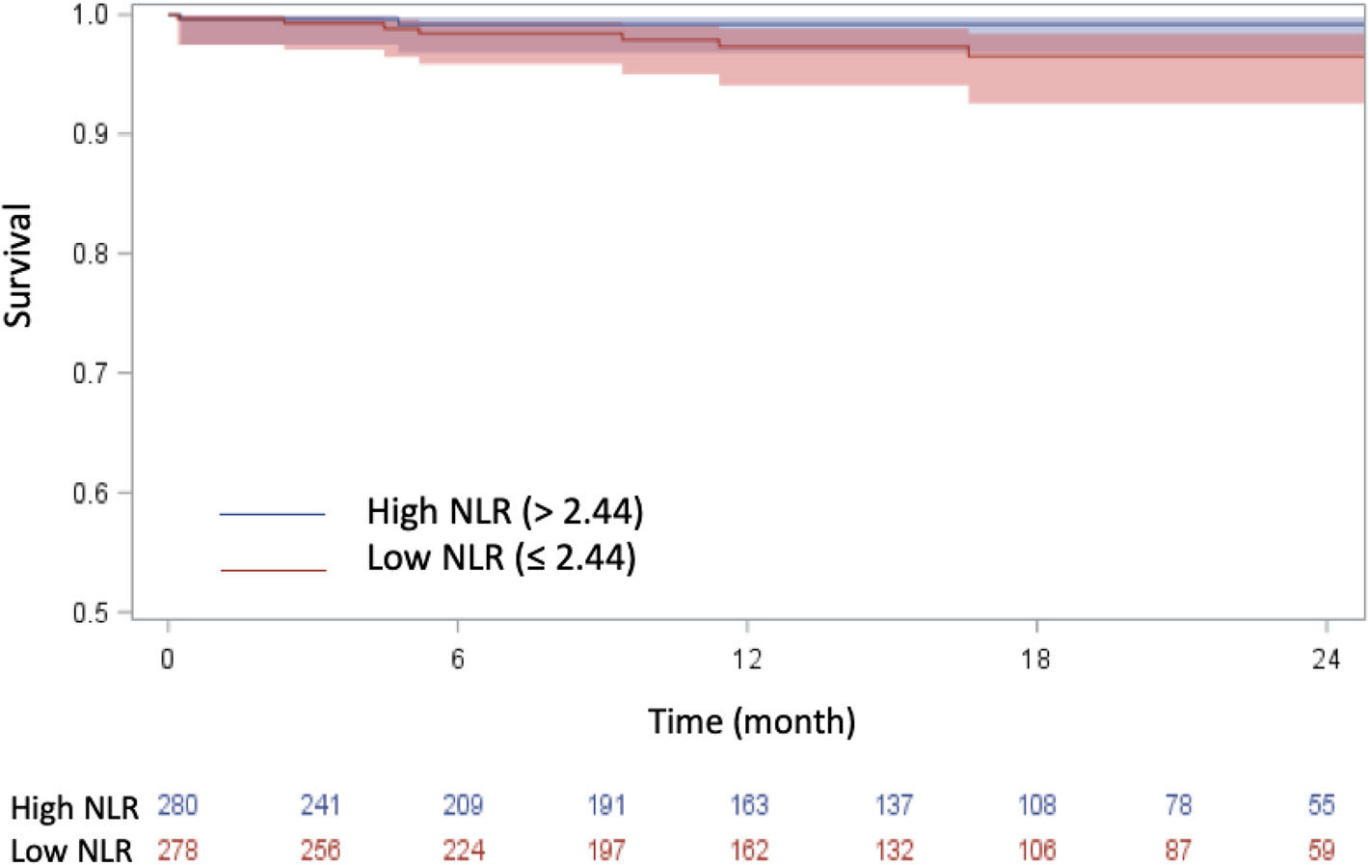
Kaplan-Meier Curve representing mid-term survival in low and high LNR cohorts Figure shows those with higher NLR appeared to have superior survival, although statistical significance was not reached

NLR and mid-term mortality had an insignificant positive association (HR = 1.33, 95%CI = .993–1.771, *p* = 0.056). LNR was inversely correlated with increased mid-term mortality (HR .001, 95%CI: 0.01–.441, *p* = .026). Data on postoperative outcomes can be seen in Table 3. A Kaplan-Meier curve comparing mid-term mortality in the high and low NLR cohorts can be seen in Fig. 2. The log rank analysis (*p* = .0656) suggests that low NLR is associated with superior survival, but the trend is not statistically significant.

## Discussion

In our study, we found LNR was a more stable measurement over time than NLR and that LNR demonstrated a stronger association with mid-term mortality. Additionally, we saw that low LNR was associated with increased baseline morbidity, increased cardiopulmonary bypass time, and increased post-operative ICU length of stay.

### Related works

Previous studies have indicated a strong correlation between the preoperative white blood cell count and cardiac death [10, 11], including studies that focused specifically on coronary artery bypass grafts [12–14]. More recent investigations have focused on the NLR as a stronger predictive measure, and several studies have demonstrated that an increased NLR is associated with complications and mortality in both cardiac and non-cardiac surgery patients. Durmus et al. demonstrated that NLR is a more predictive independent risk factor of myocardial injury following non-cardiac surgery than both of its components (absolute neutrophil count and absolute lymphocyte count) [2]. One study conducted by Tan et al. found that higher pre-operative NLR values were associated with increased risk of re-intubation (OR = 6.29, 95% CI 1.85–21.4, *P* = 0.003) and increased mortality (HR 1.85, 95% CI 1.46–2.36; *P* < 0.00001) after cardiovascular surgery [1]. Another investigation performed by Giakoumidakis et al. demonstrated increased NLR to be associated with higher morbidity including prolonged length of stay in the ICU and the hospital, delayed tracheal extubation, as well as 30-day mortality, in cardiovascular patients following cardiac surgery [15]. Gibson et al. stated that elevated NLR was found to be a strong predictor of post-operative mortality (HR 1.12 per unit, 95% CI 1.06–1.18, P b .001) among patients undergoing CABG [4]. Condado et al. found that higher baseline NLR was associated with higher preoperative risk estimations and increased occurrence of the 30-day early composite outcomes (*p* = .001, OR 1.29, 95% CI 1.04–1.61), (defined by VARC-2 to include all-cause mortality and several other post-operative complications) following transcatheter aortic valve replacement [16].

Each of these studies concluded that NLR is a significant biomarker that should be considered as part of an early risk assessment for patients being evaluated for cardiac surgery. While these studies effectively demonstrated the predictive value of NLR, they did so within a very narrow time frame. Durmus et al. and Giakoumidakis et al. only used preoperative CBC values within 24 h of the start of surgery, while Gibson et al. expanded their window to preoperative day 3. Only Silberman et al. significantly expanded this range to demonstrate the predictive value of NLR within 2 weeks of surgery. Additionally, none of the prior studies excluded patients based on immunosuppressed status or exogenous steroid use, considered the temporal stability of NLR over time, or evaluated the lymphocyte-to-neutrophil ratio as an alternative to the standard neutrophil-to-lymphocyte ratio.

### Implications

The purpose of our study was to further investigate the temporal stability of leukocyte ratios and their proposed association with post-operative outcomes in cardiac surgery, specifically focusing on patients undergoing CABG and valvular surgery. Our study is the first to our knowledge to establish the stability of NLR and LNR over time. Previous studies used relatively narrow date ranges for their analysis, which may increase the strength of NLR’s predictive value but it also limits its clinical utility. In practice, these narrow ranges limit the use of pre-operative NLR as an estimation of risk to just a few days preceding surgery. With this in mind, our study was effectively able to demonstrate NLR and LNR stability over time. While our analysis of outcomes only utilized CBC values up to 60 days prior to surgery, our study found that these values were stable up to 99 days prior to surgery. As a result, there is now evidence supporting the use of these ratios during the extended pre-operative period. This means that labs ordered throughout this period may be used to estimate risk and inform evidence-based decision making. Specifically, we found that LNR was more stable than NLR over time.

Our study was not designed or able to support the assertion that NLR values are associated with increased rates of post-operative complications or mortality. However, our study was able to demonstrate a significant relationship between LNR and mid-term mortality. Based on our data, LNR may not only be more stable, but it may also be a superior predictive measure than NLR, which has been conventionally used in previous research.

### Study limitations

Limitations to our study include single-center setting, retrospective design, and a limited sample size that yielded low incidences of complications and deaths that may have led to an underpowered analysis. Other inflammatory markers were not measured as a secondary estimate of background inflammation for comparison. Future research with a prospective design, a more comprehensive inflammatory panel, a larger sample size, and multicenter data collection is required to further investigate this topic.

## Conclusion

Our study is the first to our knowledge to demonstrate the stability of NLR and LNR values over time. While the predictive value of NLR has previously been established in the literature, our findings increase its clinical value by expanding the period of time in which it can be used to estimate pre-operative risk and inform evidence-based decisions related to patients undergoing cardiac surgery. Furthermore, LNR was demonstrated to be more stable between multiple measurements obtained under 100 days when compared with the conventionally used NLR. Low LNR was associated with decreased risk of mid-term mortality in our analysis. This may demonstrate that LNR holds greater predictive value for outcomes following valve and coronary artery bypass graft surgery, which would be a novel finding in an area of research that has solely focused on NLR.

## Data Availability

All data associated with the results is available and may be shared as requested by reviewers.

## Abbreviations

ALC: Absolute Lymphocyte Count
ANC: Absolute Neutrophil Count
AVR: Aortic Valve Repair
BMI: Body Mass Index
CABG: Coronary Artery Bypass Graft
CBC: Complete Blood Count
CHF: Congestive Heart Failure
CPB: Cardiopulmonary Bypass
eGFR: Estimated Glomerular Filtration Rate
ICC: Interclass Correlation Coefficient
ICU: Intensive Care Unit
LNR: Lymphocyte-to-Neutrophil Ratio
MVR: Mitral Valve Repair
NLR: Neutrophil-to-Lymphocyte Ration
TVR: Tricuspid Valve Repair
